# The Impact of Rubber Dam Association With Physiological Stress Parameters on Children of 7-10 Years Undergoing Pit and Fissure Sealant Treatment: An In-Vivo Study

**DOI:** 10.7759/cureus.58615

**Published:** 2024-04-19

**Authors:** Deepak Sharma, Shantanu Choudhari, Haresh Vanza, Swati Mittal, Vipin Ahuja, Nilima R Thosar

**Affiliations:** 1 Department of Pediatric and Preventive Dentistry, Government Dental College and Hospital, Ahmedabad, IND; 2 Department of Pediatric and Preventive Dentistry, Government Dental College and Hospital, Jamnagar, IND; 3 Department of Pediatric and Preventive Dentistry, Sharad Pawar Dental College and Hospital, Datta Meghe Institute of Higher Education and Research, Wardha, IND

**Keywords:** pediatric dentistry, pit and fissure sealant, stress parameters, isolation, rubber dam

## Abstract

Introduction

Anxiety is an emotion representing apprehension towards an unknown stimulus or situation. Rubber dam application during dental procedures in children makes the treatment more comfortable and acceptable as it gives them a psychological feeling that treatment is being carried out outside the oral cavity. The prime objective of this study was to evaluate and compare physiological parameters, which include pulse rate, arterial oxygen saturation level, blood pressure, and respiratory rate before and after rubber dam isolation.

Material and methods

The study consisted of 30 children patients of 7-10 years, comprised of 14 females and 16 males with a mean age of 8.15 ± 0.93 years. The study was a ‘split mouth’ clinical design study, where 60 sites in 30 patients (two sites in each patient) were used. The selected sites were divided into two groups by a convenience sampling method and were categorized as Group-I (control group - 30 sites were treated with pit and fissure sealants under cotton roll and saliva ejectors on mandibular right permanent first molar) and Group-II (study group - 30 sites were treated with pit and fissure sealants underrubber dam isolation on mandibular left permanent first molar).

Results

Rubber dam application reduced different physiological parameters of stress such as pulse rate, systolic and diastolic blood pressure, and respiratory rate at different intervals from the baseline values. Rubber dam and cotton roll applications have no significant effects on oxygen saturation levels at different intervals in healthy individuals. From statistical analysis, it was evident that a statistically significant difference was evident between the control and experimental groups (P value < 0.005).

Conclusion

It is confirmed in this study that rubber dam reduces different physiologic parameters of stress. After the application of the rubber dam, children's pulse rate, systolic and diastolic blood pressure, and respiratory rate were reduced.

Clinical significance

The study highlights the imperative role of rubber dam isolation in improving dental and medical effectiveness. In addendum to this, our research promotes the clinical use of rubber dams in pediatric dentistry.

## Introduction

Anxiety is an emotion representing apprehension towards an unknown stimulus or situation. Stress and tension are the words commonly used to simulate anxiety. Stress is also mentioned in the literature as an external response to anxiety [1]. The response is subjective; some child patients enjoy and accept dental treatment positively through self-control without displaying signs of stress. On the other side, some child patients display stress and anxiety by revealing reactions such as perspiration and excess salivation. Stress has two imperative components, physiological and psychological. Physiological stress is a reaction of a stress-inducing stimulus to physical interferences and body tissues. The psychological component is a subject-specific entity, which depends upon how a person anticipates the stimulus. Anxiety can initiate some psycho-physiological responses, which are associated with changes that occur in the cardiovascular system, sweat glands, muscles, and respiratory and digestive systems [2-4]. The clinical dental settings and protocols are anxiety-provoking and may lead to hypoxia and SpO2 drop in levels, respectively. There is a noted increase in the activity of the sympathetic branch of the autonomic nervous system. As an outcome, changes can be observed in the cardiovascular system, sweat glands, muscles, respiratory and digestive systems. Increased blood pressure, higher pulse rate, increased sweat production and muscle tone with spasms, breathlessness, dry mouth, and constipation are some of the notable signs of this activity [5,6].

Isolation during dental operating procedures is one of Barnum's most important protocols in dentistry. The work of SC Barnum in the 1860s had brought isolation with rubber dams into the picture and opened the door of its clinical application in pediatric dentistry. Later on, renowned societies of pediatric dentistry such as the British Society of Pediatric Dentistry and the American Society of Pediatric Dentistry also recommended its use for clinical dental procedures in children. Documented studies prove that even exposure to saliva for one second may form a protein layer that can resist forceful irrigation for 30 seconds, and the etching procedure needs to be repeated in these cases. Therefore, isolation for a dental procedure is an utmost requisite for the prognosis of the treatment [7-9]. One such preventive treatment procedure used in children's dentistry is sealing deep fissures with pit and fissure sealants. Pit and fissure sealant application in both primary and permanent teeth is a largely used preventive protocol to prevent decay in deep pits, grooved, and fissures in erupting permanent first molars. However, many pediatric dentists preclude its use, citing that it will be more stressful for child patients, and its application requires more time, further pushing child behavior toward the uncooperative stage [10]. The current study was carried out to evaluate the physiological parameters of child patients, which include pulse rate, arterial oxygen saturation level, blood pressure, and respiratory rate, before and after rubber dam application. Thus, we evaluated whether rubber dam isolation is stressful or not for a child.

## Materials and methods

Study area

The present study was carried out in the Department of Pediatric and Preventive Dentistry, Government Dental College and Hospital, Ahmedabad, Gujarat. The duration of the study was six months, and ethical approval for the study was obtained from the ethical committee of the same institute. The study consisted of 30 child patients of 7-10 years, comprised of 14 females and 16 males, with a mean age of 8.15 ± 0.93 years after satisfying the selection criteria. Informed consent was obtained from the parents of each subject before intervention.

Sample size estimation

Sample size estimation was done using the software G* Power (version 3.1.9.3; The G*Power Team, Germany). Based on the reference article for the unpaired t-test, the effective size was kept at 0.53, the alpha error was 5%, and the power of the study was 80%. The total minimum sample size required for the study was 29. Therefore, the sample size selected for our study was 30.

Sample selection

A total of 30 subjects within the age group of 7-10 years old were selected by a convenience sampling method. The participants were selected based on the inclusion and exclusion criteria of our research. The selection of study samples in the convenience sampling technique was based on a first-come-first-recruited basis. The children coming to the outpatient department of Pediatric and Preventive Dentistry, Government Dental College and Hospital, Ahmedabad, fulfilling the inclusion criteria were selected for the study. Thus, the convenience sampling technique is a cost-effective, simple yet effective method of sampling in research [11].

Inclusion and exclusion criteria

The inclusion criteria include healthy children with Frankl behavior rating scores of 3 and 4 [12], ages between 7 and 10 years with fully erupted mandibular first permanent molar with deep pit and fissures, and with no previous experience with rubber dams. The children excluded were subjects with Frankl behavior rating scores of 1 and 2, subjects with partially erupted or unerupted first permanent molars, subjects with latex allergy, and special children. Subjects undergoing orthodontic treatment and whose parents had not given permission for the study were also excluded.

Sample design

The study was a ‘split mouth’ clinical design study, where 60 sites in 30 patients (two sites in each patient) were used. The selected sites were divided into two groups by the convenience sampling method, and the sample was classified into two groups as follows:

Group I (control group): 30 sites were treated with pit and fissure sealants under cotton roll and saliva ejectors on the mandibular right permanent first molar.

Group II (study group): 30 sites were treated with pit and fissure sealants under rubber dam isolation on the mandibular left permanent first molar.

It was decided that the mandibular left permanent first molar would serve as the experimental group site and the mandibular right permanent first molar would serve as the control group site. In order to prevent methodological bias, it was decided that the control group site would be recorded first and then followed by the study group site, respectively. 

Data collection

During the first visit, an oral examination was carried out, and the purpose of the study with the apparatus being used was explained to the parents and the subjects. Informed and written consent was obtained from the parents of participating children. Assessment of the pit and fissure was carried out, and cleaning of the fissure was carried out with prophylaxis paste. After cleaning of fissures, etching was done by 37% orthophosphoric acid for 30-60 seconds. After etching, etchant was rinsed with water spray for 5-10 seconds, the cotton roll was changed, and the tooth was dried. After drying, fissure sealant (EmbraceTM wetbond, fluoride-releasing pit and fissure sealant) was applied, and it was light cured for 40 seconds. Physiological parameters, which include pulse rate, arterial oxygen saturation level, systolic and diastolic blood pressure, and respiratory rate, were recorded at the following intervals: (1) before the start of the treatment on the dental chair, (2) at the time of placement of cotton rolls/saliva ejectors, (3) after the placement of the pit and fissure sealant in the mandibular right first permanent molar, and (4) after the removal of cotton rolls/saliva ejectors.

Now, a rubber dam was placed in the mandibular arch, and the same pit and fissure sealant was applied on the mandibular left permanent first molar. All the steps were the same as those of cotton roll groups; except in the study group, the fist step was the administration of topical local anesthesia. The topical anesthetic gel (Lignospan-O, Septodont, France) was applied on the marginal gingiva where the rubber dam clamp was approximated to avoid discomfort to the subjects. A regular rubber dam (Coltene Hygenic Rubber Dam, USA) was used, and no effects on the gingiva and teeth were reported by the subjects while using rubber dam clamps. After the application of topical anesthesia, an appropriate size rubber dam clamp was selected, and its placement was done on the mandibular left permanent first molar. A 6x6-inch latex rubber dam sheet was selected. After that, the rubber dam sheet was inverted on the desired tooth, and the desired parameters were assessed at different intervals as follows: (1) before the start of the treatment on the dental chair, (2) at the time of placement of the rubber dam, (3) after the placement of the pit and fissure sealant, and (4) after the removal of the rubber dam. The pulse rate and arterial oxygen saturation level were assessed with the help of a fingertip pulse oximeter (Schiller Argus OMX Plus, India). Systolic and diastolic blood pressure were assessed with the help of an automatic electronic sphygmomanometer device (Citizen, Japan). The respiratory rate was assessed by the manual palpation method. All the parameters are recorded by the co-investigator and investigator at different intervals. The inter-examiner reliability was calculated using kappa statistics with a value of 0.9. Therefore, a strong agreement was noted in the findings of both examiners.

Statistical analysis

All data were encoded and compiled into a computer database in Microsoft Excel (2013 version; Microsoft® Corp., Redmond, WA). The data obtained were statistically analyzed using one-way ANOVA by Statistical Product and Service Solutions (SPSS, version 20.0; IBM SPSS Statistics for Windows, Armonk, NY) software (p-value <0.05 was considered significant).

## Results

The study consisted of 30 child patients of 7-10 years, comprised of 14 females and 16 males, with a mean age of 8.15 ± 0.93 years. All 30 children had participated in the study, and none had been excluded from the study.

Table 1 shows the pulse rate-wise distribution between the study group (rubber dam) and the control group (cotton roll) at different intervals. A paired t-test for statistical analysis was applied. The P value is >0.05 at point one, and it shows that, statistically, no significant difference was present in pulse rate between the groups at point one. P value is ≤0.05, and it shows that, statistically, a significant difference was present in the pulse rate between the groups at points two, three, and four (study group: 98.60 ± 6.22, 97.20 ± 6.73, 96.73 ± 6.66, respectively; and control group: 102.57 ± 6.74, 104.07 ± 7.62, 103.37 ± 5.81, respectively).

**Table 1:** Pulse rate-wise (units per minute) distribution between the study group (rubber dam) and the control group (cotton roll) at different intervals Statistical test: one-way ANOVA NS: Non-significant, S: Significant

Time point	Study group			Control group			P value
	Time	Mean (units per minute)	SD	Time	Mean (units per minute)	SD	
One	Before rubber dam application	100.27	6.15	Before cotton roll application	100.20	7.31	> 0.05 NS
Two	After the rubber dam application	98.60	6.22	After cotton roll application	102.57	6.74	≤ 0.05 S
Three	After sealant application	97.20	6.73	After sealant application	104.07	7.62	≤ 0.05 S
Four	After the removal of the rubber dam	96.73	6.66	After the removal of the cotton roll	103.37	5.81	≤ 0.05 S

Table 2 shows the systolic blood pressure (SBP)-wise distribution between the study group (rubber dam) and the control group (cotton roll) at different intervals. The P value is >0.05, and it shows that, statistically, no significant difference was present in the SBP between the groups at point one. The P value is ≤0.05, and it shows that, statistically, a significant difference was present in the SBP between the groups at points two, three, and four (study group: 104.40 ± 4.50, 103.53 ± 4.59, 103.20 ± 4.62, respectively; and control group: 106.43 ± 4.71, 106.17 ± 4.09, 106.17 ± 4.09, respectively).

**Table 2:** Systolic blood pressure-wise (mmHg) distribution between the study group (rubber dam) and the control group (cotton roll) at different intervals Statistical test: one-way ANOVA NS: Non-significant, S: Significant

Time point	Study group			Control group			P value
	Time	Mean (mmHg)	SD	Time	Mean (mmHg)	SD	
One	Before rubber dam application	104.97	4.65	Before cotton roll application	105.67	4.61	> 0.05 NS
Two	After the rubber dam application	104.40	4.50	After cotton roll application	106.43	4.71	≤ 0.05 S
Three	After sealant application	103.53	4.59	After sealant application	107.03	4.47	≤ 0.05 S
Four	After the removal of the rubber dam	103.20	4.62	After the removal of the cotton roll	106.17	4.09	≤ 0.05 S

Table 3 shows the diastolic blood pressure (DBP)-wise distribution between the study group (rubber dam) and the control group (cotton roll) at different intervals. The P value is >0.05, and it shows that, statistically, no significant difference was present in the SBP between the groups at point one. The mean DBP was lower in the study group than that of the control group at measuring point two. The P value is ≤0.05, and it shows that, statistically, a significant difference was present in the DBP between the groups at points two, three, and four (study group: 65.40 ± 3.59, 64.03 ± 3.73, 63.83 ± 3.83, respectively; and control group: 68.77 ± 3.92, 69.40 ± 3.40, 69.23 ± 2.78, respectively).

**Table 3:** Diastolic blood pressure-wise (mmHg) distribution between the study group (rubber dam) and the control group (cotton roll) at different intervals Statistical test: one-way ANOVA NS: Non-significant, S: Significant

Time point	Study group			Control group			P value
	Time	Mean (mmHg)	SD	Time	Mean (mmHg)	SD	
One	Before rubber dam application	67.13	4.34	Before cotton roll application	67.53	3.56	> 0.05 NS
Two	After the rubber dam application	65.40	3.59	After cotton roll application	68.77	3.92	≤ 0.05 S
Three	After sealant application	64.03	3.73	After sealant application	69.40	3.90	≤ 0.05 S
Four	After the removal of the rubber dam	63.83	3.83	After the removal of the cotton roll	69.23	2.78	≤ 0.05 S

Table 4 shows the oxygen saturation-wise distribution between the study group (rubber dam) and the control group (cotton roll) at different intervals. The P value is >0.05 at all measuring intervals, and it shows that, statistically, no significant difference was present in oxygen saturation between the groups at points one, two, three, and four.

**Table 4:** Oxygen saturation-wise (%) distribution between the study group (rubber dam) and the control group (cotton roll) at different intervals Statistical test: one-way ANOVA NS: Non-significant, S: Significant

Time point	Study group			Control group			P value
	Time	Mean (%)	SD	Time	Mean (%)	SD	
One	Before rubber dam application	98.50	0.57	Before cotton roll application	98.60	0.56	> 0.05 NS
Two	After the rubber dam application	98.53	0.50	After cotton roll application	98.33	0.80	> 0.05 NS
Three	After sealant application	98.60	0.49	After sealant application	98.47	0.50	> 0.05 NS
Four	After the removal of the rubber dam	98.60	0.49	After the removal of the cotton roll	98.37	0.66	> 0.05 NS

Table 5 shows the respiratory rate-wise distribution between the study group (rubber dam) and the control group (cotton roll) at different intervals. The P value is >0.05 at point one, and it shows that, statistically, no significant difference was present in the respiratory rate between the groups at point one. The P value is ≤0.05, and it shows that, statistically, a significant difference was present in the respiratory rate between the groups at points two, three, and four (study group: 24.40 ± 3.37, 23.60 ± 3.60, 22.80 ± 3.14, respectively; and control group: 27.37 ± 3.06, 28.53 ± 2.62, 27.13 ± 3.39, respectively).

**Table 5:** Respiratory rate-wise (units per minute) distribution between the study group (rubber dam) and the control group (cotton roll) at different intervals Statistical test: one-way ANOVA NS: Non-significant, S: Significant

Time point	Study group			Control group			P value
	Time	Mean (units per minute)	SD	Time	Mean (units per minute)	SD	
One	Before rubber dam application	26.10	3.64	Before cotton roll application	25.87	3.72	> 0.05 NS
Two	After the rubber dam application	24.40	3.37	After cotton roll application	27.37	3.06	≤ 0.05 S
Three	After sealant application	23.60	3.60	After sealant application	28.53	2.62	≤ 0.05 S
Four	After the removal of the rubber dam	22.80	3.14	After the removal of the cotton roll	27.13	3.39	≤ 0.05 S

## Discussion

A rubber dam was discovered in dentistry by S.C. Barnum in the 1860s. It has so many advantages; for example, it provides excellent isolation during dental procedures and moisture control and prevents inhalation of aerosols and debris and ingestion of foreign materials. Rubber dams are also less stressful for child patients as well as operators. Cotton rolls and saliva ejectors are more common and routine aids used for isolation in dentistry. They have several disadvantages; for example, they do not provide an accurate moisture-free environment; in addition to this, they do not provide safety from inhalation of foreign body particles and inhalation of aerosols. Some authors believe that the placement of cotton rolls in the oral cavity, especially lingual mucosa, will lead to manipulation of the floor of the mouth and this will be stressful to a child [7,13]. Our research was intended to evaluate the impact of rubber dam association with varied physiological parameters of stress such as pulse rate, oxygen saturation level, SBP and DBP, and respiratory rate during pit and fissure sealant application in children. As a standardized dental procedure, sealant application was selected because it is non-invasive and provides a comparatively lesser degree of stress to a child. The results of the present study provide evidence that rubber dam isolation is less stressful for child patients. At all the measuring intervals, different physiological parameters of stress were reduced from the baseline values. In the cotton roll group, various physiological parameters were increased at all the measuring intervals from baseline values. Both the rubber dam and cotton roll had a non-significant effect on the oxygen saturation level. Thus, from the present study, it was inferred that the child is more relaxed during isolation with a rubber dam when compared to that of a cotton roll.

In our study, the pulse rate of the rubber dam group was recorded as lesser than that of the cotton roll group. A similar result was found in a study conducted by Ammann et al. [13] where it was concluded that the pulse rate was comparatively higher in the cotton roll group than that of the rubber dam group, but the result was found statistically not significant. Contradictory results were reported in studies conducted by Myers et al. [14], Poiset et al. [15], and Bello et al. [16]. They evaluated the pulse rates during routine dental procedures in which the pulse rate was increased during the pre-injection and injection phases when compared to the pre-operative pulse rate. The pulse rate was found low during rubber dam application from the pre-injection and injection periods but was still higher than the pre-operative value. At a post-operative point, the pulse rate was decreased as compared to the pre-operative value, but the change was statistically not significant [13-16]. The possible explanation for this difference is the use of pit and fissure sealants, which is only a standardized non-invasive dental procedure, and our study did not involve any invasive dental procedures. Previous studies involved invasive dental procedures, local anesthesia administration, and placement of electrodes, which made the child anxious and, because of this, the pulse rate increased. The parameters of SBP and DBP were reported significantly lesser in the rubber dam group. Ammann et al. [13] evaluated SBP and DBP before and after isolation with cotton rolls and rubber dams. They observed that the difference was not significant with regard to the SBP and DBP in both the rubber dam and cotton roll groups. A study conducted by Bello et al. [15] found contradictory findings that a constant increase was observed in both the SBP and DBP from the preoperative baseline values, which was at zenith during the injection and post-injection phases. The value then remained greater than the baseline value during the operative period, which included rubber dam placement, cavity preparation, filling, and the post-operative period. Additionally, no significant difference was found between the average baseline blood pressure and the operative values at a significance level of p < 0.05. The possible explanation of these differences in this study could be the administration of local anesthesia and most of the patients having previous experience with local anesthesia, which might have provoked fear of eliciting a stimulus [13,16].

The respiratory rate was significantly low at all measuring intervals in the rubber dam group. Ammann et al. [13] analyzed the respiratory rate, blood pressure, and pulse rate after the isolation with rubber dam application in a study. Similar findings were observed, and statistically significant results were observed in the change of the respiratory rate in both the cotton roll and rubber dam groups at different intervals, respectively. The respiratory rate was found to be significantly lower immediately after the placement of the rubber dam. In the present study, statistically no significant difference was observed in the change of the oxygen saturation level of the rubber dam and cotton roll isolation during all measuring intervals [13]. Similar results were observed by Goodday et al. [17] who evaluated the effect of a rubber dam on the oxygen saturation level. They confirmed that the rubber dam placement did not affect the oxygen saturation levels even if it was placed properly or improperly. Similar results were also observed in studies conducted by Odabas et al. [18], Poiset et al. [15], and Bello et al. [16] where it was concluded that the values of oxygen saturation follow no specific trend. Though the values in the arterial oxygen saturation parameter displayed no significant change during the operative procedure, some insignificant desaturations were observed further down the baseline values of the pre-operative stage. The extreme decline in the values was evident during the rubber dam application. As routine dental procedures had been included in this study, inadvertent neck movement during manipulation of the oral cavity may lead to restriction of the airway and desaturation of the oxygen level [15-18]. A possible explanation of these changes in respective physiological parameters of stress may be that, after rubber dam application, children reportedly have the impression that dental treatment is being carried out outside the oral cavity. As an outcome, a significant reduction was noticed in different parameters in the rubber dam group. Another reason could be that, in rubber dam isolation, children were not supposed to spit or gargle, which led to their relaxation state as irrigation fluid did not accumulate in the oral cavity and so parameters were reduced in rubber dam isolation. The reason for the higher values of physiological parameters in cotton roll isolation could be that the placement of cotton rolls may activate the floor of the mouth, which leads to anxiety in children resulting in an increase in parameters.

Limitations of the study

The first limitation is the non-invasive treatment approach used in the study. We have used pit and fissure sealants and then analyzed the stress parameters; however, the study could have more strength if an invasive treatment approach; for example, pulp therapy would have been used. The second limitation is the small sample size. Therefore, we advise further studies with an invasive approach and a large sample size to analyze the same parameters used in our study.

## Conclusions

From the present study, it is concluded that rubber dam application reduces different physiologic parameters of stress. After the application of a rubber dam, the children’s pulse rate, SBP and DBP, and respiratory rate were reduced to significant values. During rubber dam isolation, the oxygen saturation level of children remains unaltered. After the application of a rubber dam, the children’s respiration did not deteriorate, and the oxygen saturation of the child was maintained.
